# Trichosporon asahii infection associated with glomerulonephritis in a diabetic patient

**DOI:** 10.1016/j.mmcr.2021.12.001

**Published:** 2021-12-14

**Authors:** Anandhalakshmi Subramanian, Sheela devi, Georgi Abraham, Prasanna Honnavar

**Affiliations:** a Department of Microbiology, College of Medicine, King Khalid University, Abha, Asir region, 61421, Saudi Arabia; b Department of Microbiology, Pondicherry Institute of Medical Sciences, Puducherry, 605008, India; c Department of General Medicine, Pondicherry Institute of Medical Sciences, Puducherry, 605008, India; d American University of Antigua College of Medicine, Antigua and Barbuda

**Keywords:** Trichosporon asahii, Glomerular nephritis, Diabetic patient

## Abstract

Trichosporon colonizes the skin, vagina, gastrointestinal and respiratory tract of humans. Superficial infections are common, while disseminated trichosporonosis is rare, specifically seen among immunocompromised patients and often associated with high mortality. We report a rare case Trichosporon asahii infection in a 78-year-old diabetic, with associated acute interstitial glomerulonephritis. Molecular identification of the isolate was confirmed by sequencing IGS1 region of rDNA. Our study adds to a rather limited literature on renal complications of Trichosporonosis.

## 1 Introduction

Trichosporon, a basidiomycetous yeast is a commonly found environmental and soil saprophyte. They are also known to colonize skin, vagina, gastrointestinal and respiratory tract of humans [1,2]. *Trichosporon* species have long known to be the cause of superficial infections whereas disseminated trichosporonosis is uncommon and often fatal. However, disseminated trichosporonosis is increasingly being reported from infections in the immunocompromised patients with a mortality rate of 42%–90% despite antifungal therapy [[3], [4], [5]] Most clinical isolates of this genus in the past were designated as a single species, i.e., *Trichosporon beigelii*. Over the past decade, the genus has undergone extensive taxonomic revision, which has led to identification of 50 or more species within the genus, out of which 17 are medically important and implicated in human disease. Invasive infections have been attributed to *T. asahii, T. dermatis, Trichosporon asteroides, Trichosporon inkin,* and *T. mucoides* while the other species are responsible for superficial infections [6]. Rare cases of invasive infection caused by *T. pullulans* and *T. loubieri* have been reported [7]. There are only few sporadic reports of urinary tract infections caused by *Trichosporon asahii* reported from India [8,9].We report a case of invasive *T. asahii* infection associated with glomerulonephritis in a patient with uncontrolled diabetes.

## 2 Case presentation

A 78-year-old gentleman, with long standing history of type II diabetes presented to the casualty with breathlessness and decreased urine output of 3 days duration. He had been on oral hypoglycemic drugs for the past 20 years. The patient was referred from a local hospital where he had been admitted for a period of 12 days with complaints of fever, giddiness, and dry cough for 15 days. He had been treated with injection meropenem and metronidazole for 10 days. During treatment, the patient developed signs of acute kidney injury for which he was referred to our hospital. He presented to casualty with breathlessness and decreased urine output. The day of presentation in our hospital is defined as “Day 0.”

The patient was conscious, oriented, and afebrile with a blood pressure of 170/90 mm Hg and a pulse rate of 86/min at admission. He presented with facial puffiness, bilateral pitting pedal edema, abdominal distension, and an active diabetic foot ulcer with cellulitis, measuring 15 × 7 cm in size on the medial aspect of the left leg. There was no pallor, icterus, clubbing, cyanosis or lymphadenopathy. The patient had no complaints of chest pain or loss of consciousness. He did not complain about burning micturition or hematuria. On systemic examination, the cardiovascular, respiratory and the central nervous systems were all found to be normal. Abdominal examination revealed a soft distended abdomen with sluggish bowel sounds. On investigation, liver function tests were within the normal limits, but the kidney profile revealed high levels of blood urea (162 mg/dl) and creatinine (3.4 mg/dl). He was found to be anemic with hemoglobin level of 9 g/dl. Decreased C3 (<35 mg/dl) and C4 (10.6 mg/dl) levels were also noted. There was elevated levels of C-reactive protein 24mg/dl. An Ultrasonography of the abdomen showed normal sized kidneys, a mild fatty liver, cholelithiasis and mildly enlarged prostate.

**Fig. 1:**
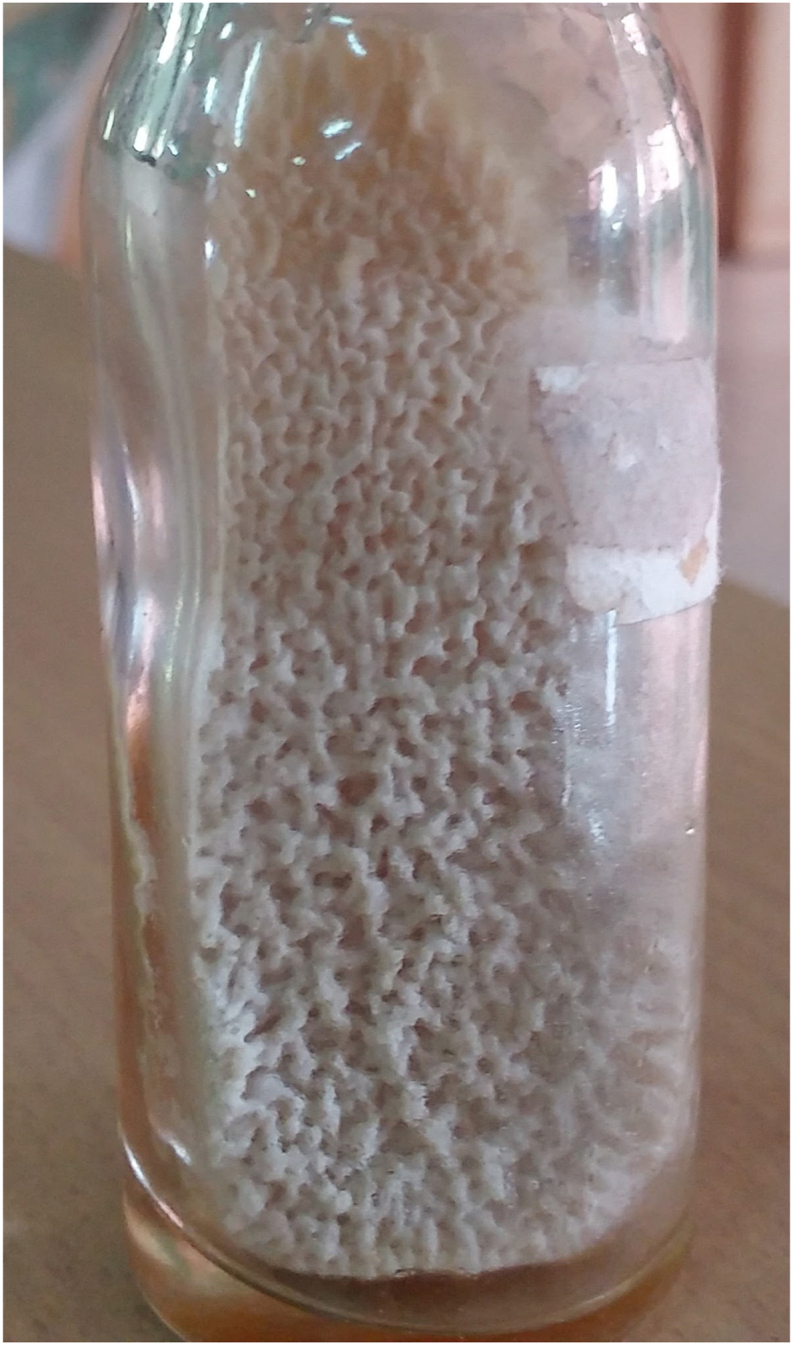
Colony morphology of Trichosporon asahii isolate on Sabouraud dextrose agar plate after 5 days of incubation at 37 °C, showing profuse wrinkled growth.

A presumptive diagnosis of acute kidney injury was made, and he was started on imipenem and clindamycin. On the day of admission “Day 0” wound swab from the site of active diabetic ulcer and midstream urine were sent for bacteriological culture and sensitivity. Direct smear of the wound swab showed few polymorphs and yeast cells. After 24 hours of incubation white, wrinkled velvety colonies with mycelial fringe was seen on sheep blood agar, Sabourauds dextrose agar and cysteine lactose electrolyte deficient agar (CLED) (Fig. 1).

**Fig. 2:**
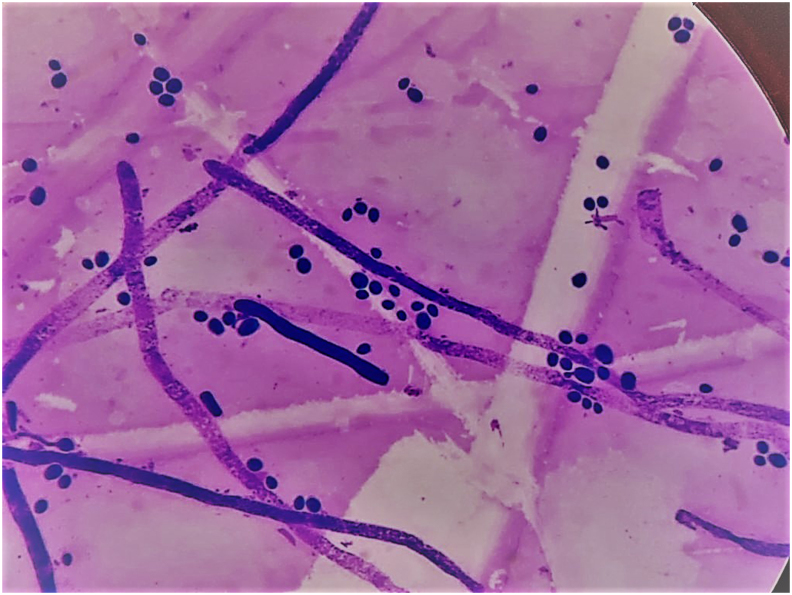
Gram stain of the colony showing septate hyphae with arthrospores.

The gram stain of the colony revealed septate hyphae with arthrospores (Fig. 2). The lacto phenol cotton blue mount showed non branching septate, hyaline hyphae and abundant rectangular arthroconidia. Based on the colony morphology and microscopy, a presumptive identification of *Trichosporon species* was made. The isolate was differentiated from Geotrichum species based on a positive urease test, ability to grow in the presence of cycloheximide and their carbohydrate assimilation pattern. Antifungal susceptibility by microdilution method as per the Clinical and Laboratory Standards Institute guidelines revealed it as sensitive to fluconazole, itraconazole and Amphotericin B with an MIC of 4,2 and 0.5 μg/ml respectively. He developed hematuria on the 2nd day of admission. A diagnosis of acute interstitial glomerulonephritis was made. Further a repeat ultrasound was performed on day 3 of admission which revealed bilateral mild hydronephrosis, and a dark hypoechogenic lesion in the right renal cortex suggestive of cortical necrosis. The patient was started on fluconazole 400mg/day, based on culture and antifungal susceptibility reports on day 3.

Molecular identification of the isolate was done by sequencing IGS1 region of rDNA.

IGS1 regions of rDNA was amplified using the primer pairs 26SF (5′-ATCCTTTGCAGACGACTTGA-3′), 5SR (5′-AGCTTGACTTCGCAGATCGG-3′) (Sigma Aldrich, Bengaluru, India) respectively. Sequencing was performed using Big Dye Terminator Cycle Sequencing Kit, version 3.1 (Applied Biosystems, CA, USA) for both strands as per the manufacturer's instructions. Sequencing reactions were purified and analysed on ABI 3130 genetic analyser (Applied Biosystems). For each gene, the consensus sequences were prepared from the sequence obtained by forward primer and reverse primers with the help of BioNumerics software version 7.1 (Applied Maths, Ghent, Belgium). The consensus sequences were compared with the sequences of the GenBank DNA database using NCBI BLAST software (http://www.ncbi.nlm.nih.gov/Genbank/index.html). The IGS1 sequence showed 100% (578/578 bp) identity with the *Trichosporon asahii* CBS2479 (accession no. FJ754250). Based on morphology and sequencing results, the isolates identification was confirmed as *Trichosporon asahii*.

However, the even after 72hrs (on day 6) of treatment with fluconazole, the CRP, urea, and creatinine remained elevated with values of 16mg/dl, 88mg/dl and 2.6mg/dl respectively.

Repeat cultures from the ulcer and two consecutive repeat urine cultures on day 7 and 14 also grew *Trichosporon asahii.* On day 11 a repeat urea and creatinine levels revealed a drop in their values (64mg/dl, 2mg/dl respectively). Hence fluconazole treatment was continued. However, on day 18, he had an episode of unstable angina and was shifted to the intensive care unit with a diagnosis of non-ST segment elevation myocardial infarction and cardiac failure for which treatment was initiated. Throughout the hospital stay, the patient did not develop neutropenia. Blood cultures on several occasions including the day of admission yielded negative results. The follow up could not be done as the patients got discharged against medical advice.

## 3 Discussion

The pathogenesis of invasive trichosporonosis primarily involves colonization of skin, mucosal surfaces of the respiratory or gastrointestinal tract followed by seeding of the bloodstream when the integrity of the mucosal surface is compromised [10]. Risk factors for haematogenous spread include long-term immunosuppressive drugs, immunocompromised status, broad-spectrum antibiotic therapy, and intravenous devices. Clinical features of disseminated infections include septic shock, pneumonia, and renal failure. Very few cases of urinary infection due to *T. asahii* has been reported earlier in the literature [11,12]. However in the past few years its incidence has significantly increased especially owing to risk factors like prolonged use of broad-spectrum antibiotics, intravenous and bladder catheterization, prosthetic cardiac valves and peritoneal dialysis [8,[13], [14], [15], [16], [17]]. Renal involvement may manifest as proteinuria and hematuria as well as renal failure [18].

In the present case, it is possible that the organism colonized the wound leading to haematogenous dissemination or colonized the urinary tract leading to an ascending infection, which may have caused the acute kidney injury resulting in hematuria and decreased urinary output.

Prior treatment with broad-spectrum antibiotics, use of a central catheter, and presence of diabetes as a comorbid condition in addition to anemia could be other possible contributory risk factors in our patient. Isolation of *T. asahii* from wound swab and consecutive 3 urine cultures and absence of any bacterial isolates from the local wound, urine, and as well as blood stream cultures establishes it as the most likely pathogen. As well the USG findings revealing bilateral hydronephrosis and right renal cortex necrosis possibly could be the complications due to disseminated renal trichosporonosis. Only a single report of renal complications due to disseminated trichosporonosis are quoted in the literature, which is interstitial tubular nephritis in a patient with primary myelofibrosis [19]. However the renal involvement has been found in most autopsies of patients with disseminated trichosporonosis [20,21].

As well in diabetic patients, there have only been few reports of disseminated trichosporonosis [8,17]. In these cases diabetes mellitus or high blood sugar was the only recognized risk factor. These cases demonstrate that *Trichosporon* sp may cause widespread dissemination and life-threatening sepsis in diabetic patients who are at risk due to chronic illness and compromised mucous membranes [8,14,17].

Trichosporonosis is now increasingly being reported both in immuno-compromised and immuno-competent individuals. It is likely to be reported as a normal flora due to lack of awareness of its potential role in infection. Risk factors such as diabetes, use of long-term broad spectrum or several antimicrobials, indwelling catheter and associated condition such as anemia and hypoalbuminemia may be the main contributing factors for establishment of opportunistic *T. asahii* infection. Microbiological evaluation of the clinical sample for fungal pathogens is a necessary to identify the agent for timely institution of appropriate antifungal therapy.

## Sources of funding

There are none.

## Consent

Written informed consent was obtained from the patient or legal guardian(s) for publication of this case report and accompanying images. A copy of the written consent is available for review by the Editor-in-Chief of this journal on request.

## Declaration of competing interest

The authors declare that they have no competing interests.
